# The improvement of mental and physical health of people with severe mental disorder: one-year efficacy of a lifestyle experimental intervention

**DOI:** 10.1192/j.eurpsy.2024.159

**Published:** 2024-08-27

**Authors:** M. Carbone, L. Mario, M. Di Vincenzo, B. Della Rocca, C. Toni, S. Cipolla, F. Martinelli, G. Sampogna, A. Fiorillo

**Affiliations:** Department of Psychiatry, University of Campania Luigi Vanvitelli, Naples, Italy

## Abstract

**Introduction:**

Patients with severe mental disorders have a significantly reduced life expectancy than the general population, often resulting from the increased prevalence of cardiovascular and metabolic diseases. Reasons include unhealthy lifestyle behaviours, reduced access to screening programs and adverse effects of many psychotropic drugs.

**Objectives:**

Our goal is to assess the efficacy of a psychosocial group intervention promoting healthy lifestyle behaviors compared to a brief psychoeducational group intervention in terms of improvement of severity of psychiatric symptoms and perceived quality of life, and a series of anthropometric and hematological parameters.

**Methods:**

This is a multicenter randomized controlled trial. Patients between 18 and 35 years of age with a diagnosis of schizophrenia and other primary psychotic disorders, unipolar depression and bipolar disorder were recruited. Exclusion criteria were inability to perform moderate physical activity, pregnancy and breastfeeding and impaired cognitive functions.

**Results:**

401 patients were recruited and randomly assigned to receive the experimental intervention (LIFESTYLE) or a behavioural control intervention. About 57% of the sample were female, with a mean age of 45.8±11.8, and BMI of 32.5±5.5. All of them were receiving almost one psychotropic drug. At one year, we observed a reduction in HOMA-IR index (from 4.3 ± 5.5 to 3.1 ± 2.9, p<0.01) and triglycerides (from 162.5 ± 78.1 mg/dL to 131.4 ± 76.0 mg/dL, p<0.001), as well as an increase in HDL (from 46.2± 14.6 mg/dL to 50.9±26.7 mg/dL, p<0.05). Moreover, a reduction in the values of BPRS “Affectivity” (from 8.7±3.0 to 7.2±2.5, p<0.001), “Activity” (from 4.7±1.9 to 4.2±1.3, p<0.01) and “Negative Symptoms” subscale (from 7.7±3.1 to 7.0±2.7, p<0.001) was also observed, along with an improvement in perceived quality of life (MANSA total score from 4.0 ± 1.0 to 5.3 ± 0.8, p<0.01).

**Conclusions:**

The results support the evidence that the LIFESTYLE intervention has long-lasting positive effects on physical and mental health of people with mental disorders. More efforts need to be done in order to increase the availability of these treatments in routine clinical settings.

**Disclosure of Interest:**

None Declared

